# Evaluation of diaphragm functions with diaphragm ultrasound and pulmonary function tests in individuals with Friedreich’s ataxia

**DOI:** 10.55730/1300-0144.5696

**Published:** 2023-09-07

**Authors:** Nur Aleyna YETKİN, Mehmet Fatih YETKİN, Burcu BARAN KETENCİOĞLU, Fatma Sema OYMAK, İnci GÜLMEZ, İnsu YILMAZ, Nuri TUTAR

**Affiliations:** 1 Department of Chest Diseases, Faculty of Medicine, Erciyes University, Kayseri, Turkiye; 2 Department of Neurology, Faculty of Medicine, Erciyes University, Kayseri, Turkiye

**Keywords:** Excursion, PImax, neuromuscular disorders, ataxia, diaphragm

## Abstract

**Background/aim:**

It is known that the correlation of pulmonary function tests (PFT) with muscle dysfunction is insufficient. Here, we aimed to evaluate the diaphragm functions in individuals with Friedreich’s ataxia (FRDA) and to examine its relationship with respiratory parameters and disease severity.

**Materials and methods:**

This prospective study, conducted between November and December 2022, at Erciyes University, included 14 individuals with genetically confirmed FRDA and an age- and gender-matched healthy control group of eight individuals. We examined pulmonary functions with spirometric methods and evaluated diaphragm excursion, and diaphragm thickness-expiratory (Tde) and – end of inspiration (Tdi) with ultrasonography during calm breathing. Thickening fraction (TF) calculated. Also, we examined PaCO2 at rest. The neurological status of individuals was assessed using the Scale for the Assessment and Rating of Ataxia (SARA).

**Results:**

The mean values of FEV1(lt), FEV1(%), FVC (lt), and FVC (%) were higher in the control group (p; <0.001, 0.013, <0.001, and 0.009, respectively). Also, mean Tdi, Tde, excursion and TF were lower in the FRDA group compared to the control group (p = 0.005, 0.294,0.005, and 0.019, respectively). The mean excursion value was 1.13 ± 0.54cm in the FRDA group and 1.71 ± 0.49cm in the control group. There is a strong, negative, and statistically significant correlation between SARA total score with excursion and TF (r = −0.7432, p = 0.002; r = −0.697, p = 0.008). There is no statistically significant relationship between excursion and BMI, standing-to-supine decrease in FVC, FEV1, and PaCO2. Also, the relationship between maximal inspiratory pressure (PImax) and excursion was moderate.

**Conclusion:**

Diaphragm ultrasound may reveal respiratory dysfunction better than PFT. Diaphragm excursion and TF are associated with disease scores in individuals with FDRA. Further studies are needed regarding the detection of alveolar hypoventilation.

## 1. Introduction

Respiratory function parameters have been used for years to examine the effects of neuromuscular diseases on respiratory muscles; however, the correlation of parameters with muscle dysfunction can be weak [1]. In recent years, there has been increasing interest in using ultrasound to help diagnose and monitor the progression of neuromuscular diseases, particularly amyotrophic lateral sclerosis (ALS). FRDA is the most common autosomal recessive ataxia [2]. The genetic abnormality results in a marked reduction of ‘frataxin’. Frataxin deficiency causes mitochondrial dysfunction, manifested by decreased adenosine triphosphate production, impaired iron-sulfur cluster assembly, abnormal iron accumulation, and generation of reactive oxygen species [3].

Neurological symptoms include gait and ataxia, dysarthria, and position dysfunction, which usually begin before age twenty-five. The most frequently identified nonneurological symptoms include diabetes mellitus, scoliosis, cardiomyopathy, and cardiac arrhythmias. Although respiratory impairment is commonly observed in individuals with FRDA in clinical practice, it is not widely documented except few studies. The studies conducted by Cisneros and Braun have shown that respiratory insufficiency and speech disorders in individuals with FDRA are associated with the impairment of the respiratory centers due to pontine and medullary atrophy [4]. In the study examining speech disorders in individuals with FDRA, the spirometric assessment revealed the presence of paradoxical movements, reduction in vital capacity, and neuromuscular respiratory insufficiency [5]. In addition, these individuals have no data on diaphragm examination yet. It has been previously reported that sleep-related breathing disorder is more common in individuals with FRDA, and obstructive sleep apnea correlates significantly with disease duration and clinical severity of FRDA [6]. The only retrospective study examining respiratory involvement in individuals with FDRA; demonstrated varying degrees of impaired respiratory function in the FRDA [7]. In that study, despite including patients under 18 with a disease duration of less than 15 years, respiratory muscle involvement was found to be 70%.

The diaphragm is the primary respiratory muscle used for breathing at rest. Diaphragm function may be affected by central nervous system diseases or neuromuscular disorders [8]. Diaphragmatic dysfunction initially manifests as exertional dyspnea; it can gradually cause alveolar hypoventilation and eventually respiratory failure requiring mechanical ventilation. An accurate and early diagnosis of diaphragm dysfunction is therefore essential to evaluate the timing for noninvasive mechanical ventilation (NIV) and obtain prognostic information on neurological disorders [9]. The aim of this study is to assess the respiratory functions of individuals with FRDA by diaphragm ultrasound and pulmonary function tests (PFT) and to compare them with healthy controls.

## 2. Materials and methods

### 2.1. Study design

This observational study was conducted by the Declaration of Helsinki and was approved by the Ethics Committee of Erciyes University (Meeting Date of the Ethics Committee: 28/09/2022, decision number: 2022/671). Erciyes University supported this work, a scientific research project, grant number [THD-2022-12428]. Individuals who were followed up with the diagnosis of FRDA and met the inclusion criteria were prospectively included in the study between November 2022-December 2022, in the pulmonology department.

### 2.2. Participants

Fourteen individuals aged 18 to 65 with a genetically confirmed diagnosis of FRDA were invited to participate in the study. Individuals with any disease other than FRDA that could affect respiratory functions were excluded. The neurological status of individuals was assessed using the Scale for the Assessment and Rating of Ataxia (SARA) which had been previously evaluated in terms of validity and reliability [10]. The SARA score assesses the severity and progression of ataxia by evaluating various motor functions, including posture, gait, kinetic, and speech. Scoring in the SARA score ranges from 0 to 40; higher scores were associated with more disability. It provides clinicians with a standardized way to measure and monitor the neurological impairment associated with Friedrich’s Ataxia, aiding in disease management, treatment planning, and assessing the effectiveness of interventions [11].

Eight age- and gender-matched healthy individuals were included as a nonblind control group.

### 2.3. Spirometric analysis and arterial blood gas analysis

FVC, FEV1, and FEV1/FVC were measured by simple spirometry (Vmax, ENCORE, Sensormedics, Yorba Linda, California, USA). Simple spirometry was repeated in the supine and sitting positions separately. The change in FVC was recorded as a percentage. A single specialized respiratory physiotherapist performed tests following the method recommended by the European Respiratory Society (ERS)/American Thoracic Society (ATS) guideline [12]. Three acceptable recordings were obtained for each spirometric parameter, and the best one was recorded.

The maximal inspiratory pressure (PImax) and maximal expiratory pressure (PEmax), used to assess respiratory muscle strength, were measured using a respiratory dynamometer (Vmax, ENCORE, Sensormedics, Yorba Linda, California, USA) following the method recommended by ERS/ATS [12]. PImax and PEmax values were expressed as percentages of the predicted values. The arterial carbon dioxide pressure (PaCO2) values were obtained with arterial blood samples taken from the radial artery between 8–9 AM.

### 2.4. Ultrasonographic diaphragm analysis

#### 2.4.1. Diaphragm excursion

**Figure 1:**
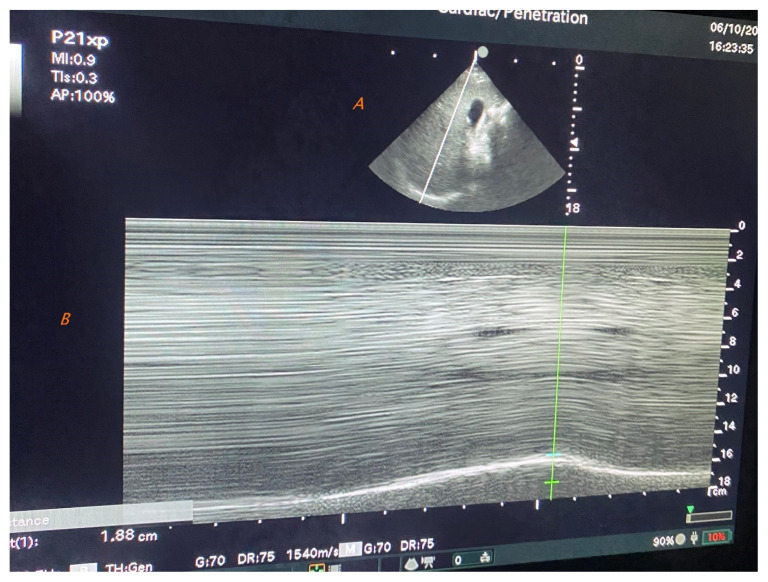
A. Measurement of diaphragm thickness in intercostal view (between midclavicular and anterior axillary line) using B mode and curvilinear transducer with liver as acoustic window. B. Measurement of right hemidiaphragm excursion in subxiphoid view using M-mode and curvilinear transducer once regular breathing waves are established. The waves appearing in the healthy diaphragm are observed to be decreased.

We used an ultrasound device (FC1, Fujifilm-Sonosite, WA, USA) to measure the indices of the diaphragm with a sector transducer(2–5MHz). Diaphragm movements were recorded within the B-Mode. For navigation, ultrasound beams were placed midclavicular into the subcostal space to visualize the diaphragm vertically and switched to M mode. The angle of the probe was adjusted so that the ultrasound beam was perpendicular to the posterior third of the right hemidiaphragm. The procedure started at the end of normal expiration and allowed subjects to inhale calmly. Measurement of diaphragm thickness and excursion with ultrasonography is shown in Figures 1A and 1B respectively.

#### 2.4.2. Thickening

**Figure 2:**
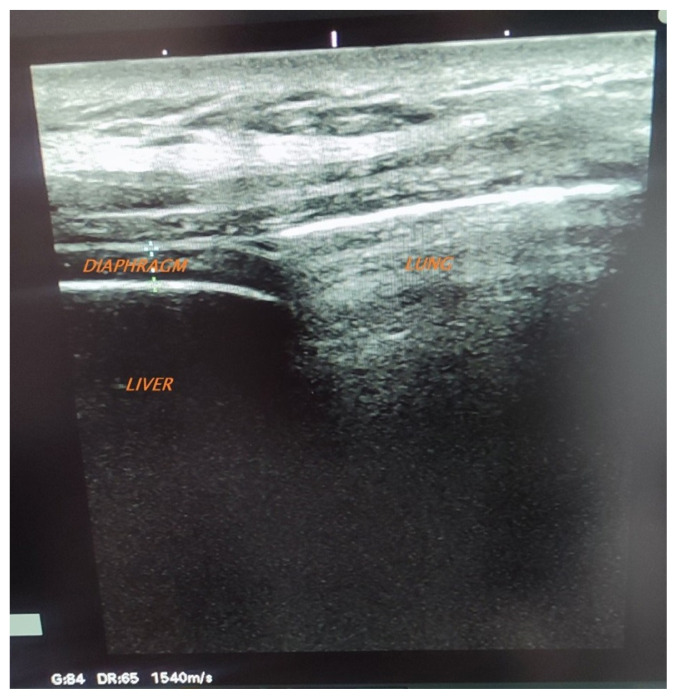
Ultrasound image of the normal right hemidiaphragm in the zone of apposition.

As shown in Figure 2, we measured the thickness of the diaphragm in the apposition zone during normal breathing using B-mode ultrasound imaging. Measurements were made in a 30–45**°** sitting position with a 6–13MHz linear transducer placed on the chest wall in the 8th–9th right intercostal space between the anterior-axillary and mid-axillary lines [13]. The thickness of the diaphragm was measured to the nearest 0.1 mm with an electronic caliper as the distance from the deep edge of the peritoneum to the superficial edge of the diaphragmatic pleura. Diaphragm thickness was measured both at the expiratory- functional residual capacity (FRC) level-Tde and the end of inspiration-Tdi during quiet breathing.

All diaphragmatic measurements were made on the right side only (12) due to constraints in the splenic acoustic window and acoustic barriers of air in the stomach and intestines on the left [14]. Each determined parameter was repeated three times and recorded, and finally, the average value was taken.

The diaphragm ultrasound was performed by a single experienced pulmonologist (N.A.Y) specialized in this field. A preliminary study was initially conducted on a group of healthy volunteers, followed by an investigation involving patients under intensive care due to respiratory failure. Before conducting the study, informed consent was obtained from all participants or their legal representatives. All ultrasonographic measurements were conducted three times, and the mean value was subsequently utilized for data analysis to ensure a representative and reliable estimation of ultrasonographic variables. A high intra-rater agreement was observed during the preliminary and the main study. The intraclass correlation coefficient (ICC) was 0.91 and 0.93 for the excursion, respectively.

#### 2.4.3. Thickening fraction

Position the patient in a comfortable semirecumbent or supine position, ensuring the diaphragm is within the ultrasound field of view. To measure the diaphragm thickness and calculate the diaphragm thickening fraction (TF), the following steps are followed: Identify a standardized anatomical landmark on the diaphragm image, such as the mid-zone. Use measuring tools like calipers or digital measuring tools to measure the thickness of the diaphragm at the identified landmark during both inspiration and expiration. Repeat the measurement for each image to ensure accuracy and reliability. The formula is used to calculate the diaphragm thickening fraction: TF = [(Inspiration thickness–Expiration Thickness)/Expiration Thickness] **×** 100. In some studies, in the literature, diaphragm TF assessments have been performed after inspiratory efforts up to the Total Lung Capacity (TLC) level [15]. In our study, since patients could not perform deep inspirations, the evaluation of TF was calculated based on diaphragm thickness measurements relative to tidal volume and FRC levels.

### 2.5. Statistical analysis

All analyses used TURCOSA (www.turcosa.com.tr, Turcosa Analytics Solutions Ltd Co, produced by Turkey). A p-value less than 0.05 was considered statistically significant. The Kolmogorov-Smirnov and Shapiro-Wilk tests were used to determine whether data is distributed normally. Mann Whitney U test was used to compare non-normal distribution data, and the results of the analysis were given as median (min-max). The Student’s t-test has been used for normally distributed data. The results of the analysis were presented as mean ± standard deviation. The Pearson chi-square test analyzed categorical variables, and the results were presented as frequency (%). Pearson correlation analysis was used for the correlation analysis of normally distributed data, while Spearman correlation analysis was used for non-normally distributed data.

## 3. Results

We enrolled fourteen consecutive individuals with FRDA and eight control participants. While 8 (57.1%) of the FRDA group were female, 4 (40 %) of the control group were female. The distribution of all data except for SARA was normal according to the Shapiro-Wilk normality test. The mean age of the FRDA group was 33.5 ± 6.9 years, and the mean age of the control group was 33.7 ± 4.8 years. There was no statistical difference between the groups regarding gender distribution and mean age (p = 0.408 and 0.938, respectively). The mean BMI was lower in the FRDA group than the control group (19.60 ± 4.9 kg/m^2^ and 26.17 ± 3.1 kg/m^2^; p = 0.001). Also, there was no difference between the groups regarding smoking status (p = 0.481). The median SARA score was 23 (14–33) in the FRDA group. The mean values of FEV1(lt), FEV1 (%), FVC (lt), and FVC (%) were higher in the control group (p = <0.001, 0.013, <0.001, and 0.009, respectively). FEV/FVC ratios were similar between the groups (p = 0.909). Similarly, mean Tdi, Tde, and excursion were lower in the FRDA group compared to the control group (p = 0.005, 0.294, and 0.005, respectively). Demographic characteristics and PFT results of individuals with FRDA and healthy controls are presented in Table.

We found the mean PImax value of the FDRA group to be 58.45 ± 16.2 cmH2O. A strong, positive, statistically significant correlation exists between PImax and PaCO2 (r = 0.6469, p = 0.031. Also, a strong, negative, and statistically significant correlation was found between PImax and standing-to-supine decrease in FVC (r = −0.6942, p = 0.018). The relationship between PImax and excursion was moderate and positively correlated (r = 0.5797, p = 0.062). No statistically significant correlation was found between PImax with Tde and SARA total score and other parameters (r = 0.3695, p = 0.263; r = −0.3372, p = 0.311 respectively).

We found a strong, negative, and statistically significant correlation between FVC in the supine position and SARA total score (r = −0.6552, p = 0.015). There is no statistically significant correlation between SARA total score and the standing-to-supine decrease in FVC (r = 0.1263, p = 0.681).

While the mean Tde value was 1.12 mm in the FRDA group, it was 1.6 mm in the control group. There was no statistically significant correlation between the Tde value and PFT, SARA total score, excursion, and PaCO2 values.

**Figure 3:**
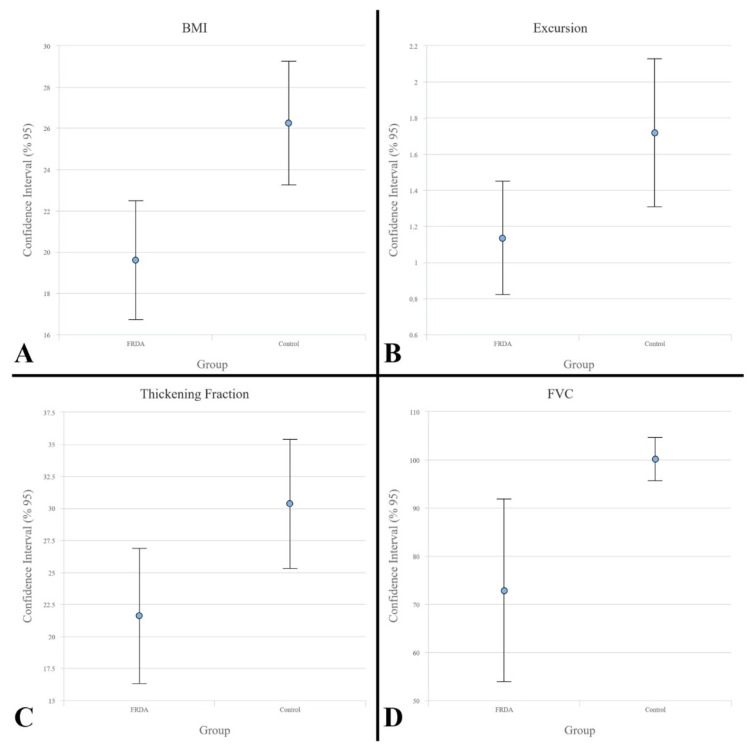
Error bar graphs for body-mass index (BMI), excursion (cm), thickening fraction (TF) (%), and force vital capacity (FVC) (%) values between individuals with FDRA and control groups.

The patient group’s mean TF value was 21.5%, while that of the control group was 33.5% (p = 0.019). There is a strong negative correlation (r = −0.6976, p = 0.008) between TF and SARA score variables, which is statistically significant. Although we found a moderate positive correlation (r = 0.493, p = 0.027) between TF and FVC variables, we did not observe a significant relationship between TF and standing-to-supine decrease in FVC, PImax (p = 0.61 and p = 0.71, respectively). The error bar graphs for BMI, excursion, TF, and FVC values between individuals with FRDA and control groups are shown in Figures 3A, 3B, 3C, and 3D respectively.

**Figure 4:**
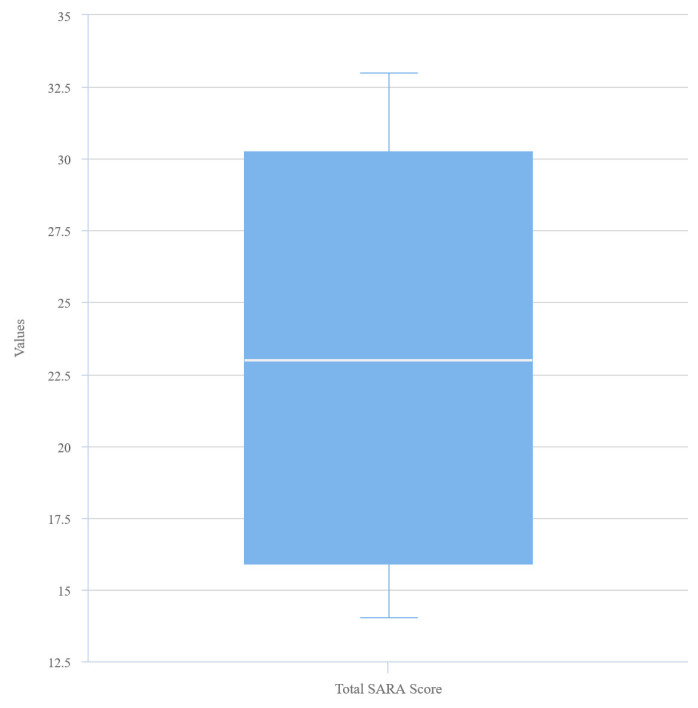
Box plot graph for SARA score.

**Figure 5:**
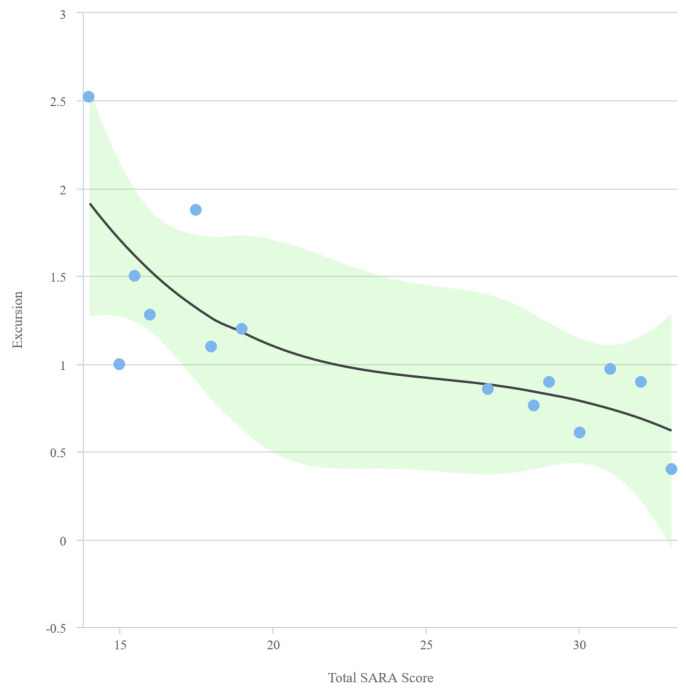
Curve of total SARA score and excursion values.

**Figure 6:**
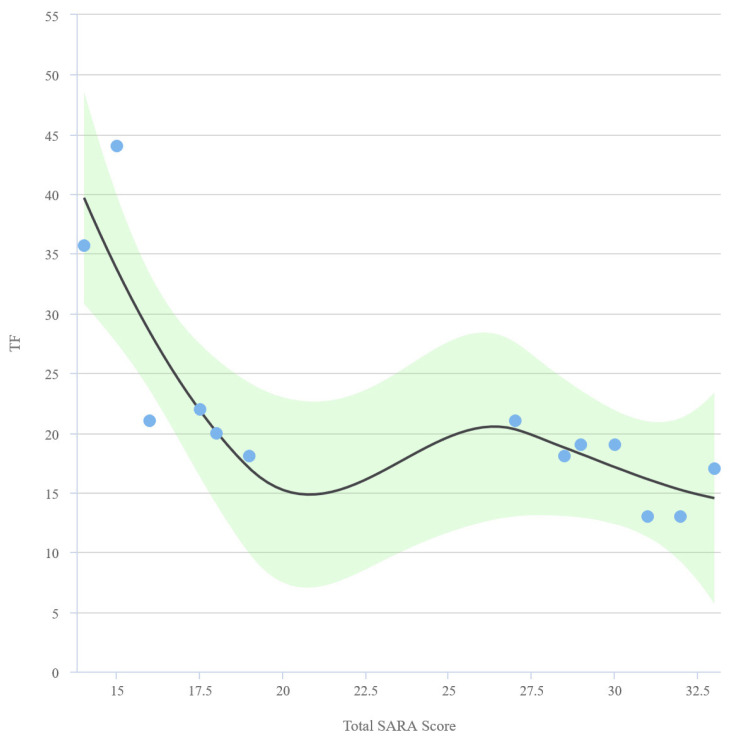
Curve of disease total SARA score and TF (%) values.

The mean excursion value was 1.13 ± 0.54 cm in the patient group and 1.71 ± 0.49 cm in the control group. A strong, negative, and statistically significant correlation exists between SARA total score and excursion (r = −0.7432, p = 0.002). A scatter plot graph representing the relationship between excursion and SARA score is shown in Figure 4. No statistically significant relationship exists between excursion and BMI, standing-to-supine decrease in FVC, FEV1, and PaCO2. The curves of SARA total score, excursion, and TF (%) values are displayed in Figures 5 and 6, respectively.

## 4. Discussion

This is the first study to evaluate pulmonary function with diaphragmatic ultrasound in individuals with FDRA. In this study, we found that the diaphragm excursion and TF values correlated with SARA score. Although the diaphragm thickness decreased in these patients, it did not show a relationship between the severity of the disease and other respiratory function parameters.

PImax and PEmax, which are static pressure measurements, reflect the maximum pressure generated by the respiratory muscles against an occluded airway and the elastic recoil pressure of the lung and chest wall in the case of airway obstruction. The PImax and PEmax measurements assess overall respiratory muscle function rather than specific muscles. A PImax in the normal range may help exclude significant respiratory muscle dysfunction, but abnormal results may be due to poor test performance rather than true respiratory muscle weakness [16]. It is recommended to use PFT in combination with variable predictors in respiratory muscle evaluation [1]. Nocturnal capno-oximetry can be used to assess nocturnal hypoventilation or can be evaluated by polysomnography [17]. For these reasons, ultrasonographic evaluation of the diaphragm has become widespread due to its ease of use and transportation. Studies about the diaphragm in neuromuscular diseases and critical patients have become widespread.

The diaphragm thickness averages 1.6 to 3.4 mm when healthy subjects rest at FRC [18]. Diaphragm thickening measurements obtained by ultrasonography are angle dependent. In addition, especially with the deep inspiration maneuver, the diaphragm may move to the “line of sight” of other areas, and the image may be distorted. The extent of diaphragmatic thickening for a given level of inspiratory effort varies considerably between subjects [19]. In our study, the mean value of Tde was 1.12 mm, which we found significantly lower than the control group. Studies have shown that the diaphragm thickness is 1.50 mm in patients with diaphragmatic weakness [20, 21]. In our study, the low Tde value in the control group and compared to the literature indicates diaphragm weakness in these patients.

Nevertheless, which value of Tde can be used to determine diaphragmatic dysfunction still needs to be determined. Another study reported that diaphragm functions and weaning success, as confirmed by esophageal pressure measurement in ICU patients, are not correlated with Tde [22]. It has been shown that Tde is not significantly correlated with respiratory muscle involvement with or without diaphragmatic weakness. Diaphragm thickness is significantly reduced in ALS patients compared to healthy controls [23, 24]. Studies are reporting that the thickness is negatively correlated with PaCO2, especially the thickening ratio [23]. Although diaphragm thickness in deep inspiration was also associated with PEmax, sniff nasal inspiratory pressure (SNIP), and FVC, in another follow-up study, thickening, and FVC did not decrease. Our study, like the existing literature, did not identify any association between Tde and any respiratory parameters.

In unilateral diaphragmatic weakness, a 10%–20% decrease in FVC can be observed in the supine position compared to the sitting position. In severe bilateral diaphragm weakness, FVC is usually approximately 50% predicted and can further decrease by 30% or more when supine [25]. In a study examining the change in bilateral diaphragmatic dysfunction on the FVC, the difference was observed between 10%–47% [26]. In the only study performed on patients with FDRA, no significant change in the standing-to-supine decrease in FVC [7]. However, we found an average of 8% (4.1%–13.8%) change. However, this is a change that can be seen in normal healthy individuals, and it was not correlated with disease severity. The fact that the average value is low may be due to poor reflection of the diaphragm function of the FVC.

PImax is strongly related to exertional dyspnea. The percentage of predicted value indirectly reflects inspiratory muscle strength. However, some studies have shown that PImax is a poor predictor of nocturnal desaturation [27, 28]. In unilateral or bilateral diaphragmatic paralysis, it can be expected to reduce PImax by <60% of the predicted values. In our study, mean PImax was found to be 59.66%. This may be an indication of diaphragmatic weakness that can develop in individuals with FDRA. However, PImax may also change if there is structural lung disease [29].

Another study conducted on ALS patients reported that the SNIP test is a better parameter than FVC and PImax in predicting alveolar hypoventilation [30]. In our study, although PImax did not reflect the severity of the disease, it showed a severe relationship between CO2 and FVC change. Still, no association was found between PImax and the total SARA score, Tde. It may be due to the low sensitivity of PImax in showing diaphragmatic weakness alone. Also, the moderate correlation between PImax and excursion in our study may be attributed to PImax better reflecting the global muscle condition and showing less specificity to diaphragm functions.

The excursion of the right diaphragm is the parameter with the highest reliability among practitioners in terms of ease and clarity. Also, the healthy population’s mean excursion value was 1.8 ± 0.3 cm at tidal volume [31]. In another study in healthy individuals, the mean value of right diaphragm excursion during normal breathing was found to be 2.32 ± 0.54cm [32] and it was emphasized that values below this could be used in the context of atrophy. Also, diaphragm excursion changes when the breathing pattern changes. It is generally recommended to be measured with the quiet breath order and voluntary sniffing maneuver [31]. Since most of our patients could not perform deep inspiration, we made the excursion measurements in the study at tidal volume. It has previously been shown to be effective in identifying diaphragmatic weakness, COPD exacerbation, and cerebral infarction [33, 34]. The cutoff values provided in studies related to diaphragm dysfunction are variable. Values ranging from 1.0 to 1.5 cm in tidal volume have been reported for excursion, indicating diaphragm dysfunction. In our study, an excursion value of 1.13 cm was found, which may reflect diaphragm dysfunction. Also, we found that excursion was moderately positively correlated with PImax, consistent with the literature. We also determined that excursion was strongly associated with the SARA score.

Excursion value can also change with age, gender, body composition, and scanning position [35, 36]. In our study, the BMI of individuals with FDRA was found to be low. The low excursion value in our study may be due to low BMI and the high female gender in the patient group, apart from the severity of the disease. In addition, a statistically significant negative correlation was found between age and excursion. Also, a statistically significant negative correlation has been reported between age and excursion [32]. However, in our study, the patient and control groups did not comprise advanced age.

The diaphragm TF reflects variation in the thickness of the diaphragm during respiratory effort. The excursion, which is associated with inspiratory volume, is not correlated with other indexes of respiratory effort. On the other hand, TF reflects more of the active diaphragm contraction. In previous studies, the TF value in healthy individuals has been found to be between 30%–36% [37–39]. It has been reported that the lower limit of normal for TF in healthy subjects and patients with COPD is >20% [37, 40]. The variability of values in studies regarding TF measurement is attributed to variations in measuring at the TLC level and normal tidal volume level. In our study, since individuals with FDRA do not have the respiratory muscle strength to breathe until the TLC level, TF measurement was calculated based on the tidal volume level. In our study, the average TF value in the patient group was found to be 21%, which was significantly lower compared to the control group. The reason for being lower than the reported cut-off value of 20% for diaphragm dysfunction in the literature may be due to the limited number of patients in the study. The moderate correlation observed between TF and FVC values may suggest that FVC is more specific to diaphragm functions compared to PImax.

There are limitations to this study that must be addressed. First, this was a single-center and nonblind study with a relatively small number of participants. However, we did not include the left diaphragm in the evaluation, although evaluation of the left hemidiaphragm without diaphragmatic lesions or palsy did not change the results of this study. Another limitation of our study is the lack of evaluation of TLC and residual volume (RV) using plethysmography. An increased RV/TLC value in the absence of obstruction and a decreased TLC value can also be indicative of respiratory muscle weakness [16].

Also, we did not evaluate patients’ nocturnal hypoventilation with nocturnal oximetry or polysomnography. Data on the increased incidence of obstructive sleep apnea disorder in individuals with FDRA are limited in the literature. Further studies are needed to evaluate nocturnal hypoventilation in individuals with FDRA.

## 5. Conclusion

PFTs are not sufficient to evaluate the function of the respiratory muscles. Diaphragm ultrasound may reveal respiratory dysfunction better than pulmonary function tests in individuals with FDRA. Diaphragm excursion and TF are associated with disease severity.

## Figures and Tables

**Table:** Demographic characteristics and pulmonary function tests of people with FRDA and healthy controls.

		FRDA (n = 14)	Control (n=8)	p
Age (year)		33.5 ± 6.9	33.7 ± 4.8	0.938*
Gender n, (%)	Female	8 (57.1)	4 (40)	0.408#
	Male	6 (42.9)	6 (60)	
BMI (kg/m2)		19.60 ± 4.9	26.17 ± 3.1	0.001*
SARA score		23 (14–33)	-	
Smoking status n, (%)	Nonsmoker	11(78.6)	7(70)	0.481#
	Smoker	2(14.3)	3(30)	
	Ex-smoker	1(7.1)	0(20)	
PFT in sitting position	FEV1(lt)	2.45 ± 0.9	4.17 ± 0.8	<0.001*
	FEV1(%)	73.64 ± 30.3	99.9 ± 4.5	0.013*
	FVC (lt)	2.87 ± 0.9	4.51 ± 0.8	<0.001*
	FVC (%)	73.2 ± 27.6	98.90 ± 6.5	0.009*
	FEV/FVC ratio	84.91 ± 11.6	85.35 ± 2.7	0.909*
PFT in supine position				
FVC (lt) FVC (%)FVC (lt) 67.28 ± 28.3		2.57 ± 0.9	-	-
		-	-	
Delta FVC		8.39 ± 7.5	-	-
PImax		58.45 ±16.2	-	-
PImax (%)		59.66 ± 18.4	-	-
PEmax		53.7 ± 21.5	-	-
Tdi (cm)		0.18 ± 0.06	0.20 ± 0.03	0.005*
Tde (cm)		0.11 ± 0.04	0.16 ± 0.03	0.294*
TF (%)		21.5 ±8.7	33.5 ±6.03	0.019*
Excursion (cm)		1.13 ± 0.5	1.71 ± 0.49	0.005*

PFT: Pulmonary function test, FEV1: Forced Expiratory Volume in 1 s, FVC: Forced vital capacity, Delta FVC: Standing-to-supine decrease in FVC, PImax: Maximal inspiratory pressure, PEmax: Maximal expiratory pressure, TF: Thickening fraction Tde(cm): Diaphragm thickness at the expiratory- at FRC level, Tdi(cm): Diaphragm thickness at end of inspiration during quiet breathing: Values are presented as mean ± standard deviation for normally distributed data, median (minimum-maximum) for no-normally distributed data and n (%) for categoric variables.

* Student’s t test,

# Pearson chi-square test
